# Comparative Metabolomic and Transcriptomic Analysis of Flavonoid Accumulation in the Zest and Pith of Guang Foshou at Green and Ripe Stages

**DOI:** 10.3390/foods15213946

**Published:** 2026-11-03

**Authors:** Wei Sun, Xuehang Wu, Wenlin Xu, Jiyan Chen, Qijie Liu, Nasir Abdullah, Xiaorong Wan, Fengmin Liu, Yunling Gao, Lihong Zhai, Weili Zhang

**Affiliations:** 1 College of Agriculture and Biology, Zhongkai University of Agriculture and Engineering, Guangzhou 510225, China; starking521@126.com (W.S.); 15875684344@163.com (X.W.); jetliu.233@gmail.com (Q.L.); biowxr@zhku.edu.cn (X.W.); fmliu68@163.com (F.L.); 2 Deqing Zhongkai Agricultural Industry Technology Innovation Research Co., Ltd., Zhaoqing 526639, China; qezcbmp3@163.com; 3 Institute of Neuroscience and Brain Science, Xiangyang Central Hospital, Affiliated Hospital of Hubei University of Arts and Science, Xiangyang 441053, China; 2023168181@hbuas.edu.cn (W.X.); an9610902@gmail.com (N.A.); gylfighting@hbuas.edu.cn (Y.G.); 4 Zhaoqing Agricultural Demonstration and Promotion Center for Improved Varieties, Zhaoqing 526040, China

**Keywords:** *Citrus medica* ‘Fingered’, flavedo, albedo, flavonoid, spatiotemporal pattern, metabolome, transcriptome

## Abstract

*Citrus medica* ‘Fingered’ (known as Guang Foshou in Chinese) is a distinctive edible fruit and a traditional herbal medicine enriched with bioactive flavonoids. However, the spatiotemporal patterns of flavonoid accumulation in the zest (flavedo) and pith (albedo) throughout fruit development remain poorly characterized. This study combined metabolomic and transcriptomic analyses to examine green and yellow fruits. Total flavonoid content increased significantly in the zest during fruit ripening, rising from 1.60 to 2.46 mg/g, whereas it remained relatively stable in the pith at approximately 2.2–2.3 mg/g. In the pith, flavanone glycosides, including hesperidin, neohesperidin, and naringin, accumulated progressively and are recognized for their cardioprotective and anti-inflammatory properties. In comparison, polymethoxylated flavones such as nobiletin and tangeretin, together with the methylated flavonol syringetin, accumulated in the zest, contributing to fruit coloration and showing well-known anti-inflammatory and anticancer activities. Transcriptomic analysis revealed that the flavonoid biosynthesis pathway involves core structural genes, including *CHS*, *CHI*, *FLS*, *CYP75B2*, *FGT*, and *UFGT*, which play crucial roles in regulating tissue-dependent accumulation patterns. Pathway analysis further showed that plant hormone signal transduction and carotenoid biosynthesis were enriched in the zest, with ethylene biosynthesis (*ACO1*) and abscisic acid biosynthesis (*NCED2* and *CCD4*) genes upregulated during the green-to-yellow transition and co-expressed with flavonoid structural genes. We propose a two-tier system of quality markers for Guang Foshou: diosmin, which did not differ between the zest and the pith (q = 0.32) but declined by 22% from the green to the ripe stage (q = 0.0029), is suitable as a tissue-independent baseline marker provided that it is referenced to a defined maturity stage, whereas syringetin (67-fold higher in ripe zest than in ripe pith), 5-demethylnobiletin (43-fold) and the flavanone glycosides hesperidin, neohesperidin and naringin, which were strongly enriched in the pith, can discriminate tissue origin and ripening stage. This tiered framework provides a scientific basis for medicinal quality control and molecular breeding of Guang Foshou.

## 1. Introduction

*Citrus medica* ‘Fingered’, commonly known as Guang Foshou in Chinese, is a citrus fruit in the family Rutaceae [1]. It is cultivated predominantly in the Guangdong and Guangxi Provinces of China. It is characterized by a golden-yellow color, elongated, slender, finger-like segments, and a pleasant aroma. Guang Foshou may be consumed fresh or dried for use as a traditional herbal remedy. It contains various bioactive components, including flavonoids, neolignans, coumarins, carotenoids, polysaccharides, polyphenols [2], terpenes, and amino acids. Experimental evidence has shown that this fruit possesses anti-inflammatory [3,4], antimicrobial [5], and hypolipidemic [3,4] activities.

Among these biologically active compounds, flavonoids, historically referred to as vitamin P, represent a major class of plant-derived secondary metabolites with numerous health-promoting properties and both preventive and therapeutic effects. Their reported biological activities include antioxidant [6], anti-inflammatory [7], cardioprotective [8], anticancer [9], neuroprotective [10], antimicrobial and antiviral effects [11] together with metabolic regulation [12], immunomodulation [13], and cutaneous protection [14]. Approximately 4000 flavonoids have been isolated. These compounds represent a chemically diverse family of polyphenolic molecules characterized by a phenyl-benzopyran (C6-C3-C6) structural backbone and are widely distributed throughout plants. Based on the linkage position between the aromatic B ring and the benzopyran C ring, flavonoids are classified into flavonoids, isoflavonoids, neoflavonoids, and minor flavonoids. Flavonoids contain a 2-phenyl-benzopyran structure and include flavanones, dihydroflavonols, flavanols, flavones, flavonols, and anthocyanidins. Isoflavonoids possess a 3-phenylbenzopyran structure and include isoflavonols, isoflavones, isoflavonoids, and pterocarpans. Neoflavonoids contain a 4-phenyl-benzopyran structure and include 4-arylcoumarins, neoflavans, and 3,4-dihydro-4-arylcoumarins. Minor flavonoids include 2′-hydroxychalcones, 2′-hydroxydihydrochalcones, aurones, and auronols [15]. In plants, these compounds perform essential physiological and ecological functions, particularly in defense responses, pigmentation, and protection against ultraviolet radiation. Citrus fruits are generally abundant in flavones and flavanones, along with associated dihydroflavonols and anthocyanins [16]. Foshou contains many phenolic compounds, with flavonoids being the predominant group. Compounds such as hesperidin, scoparone, scopoletin, 5,7-dimethoxycoumarin, and chrysoeriol-7-O-glucoside have been involved in regulating insulin resistance and have been used in interventions targeting hyperlipidemia and atherosclerosis. Several studies have consistently identified diosmin and hesperidin as the predominant flavonoids of Foshou across geographical origins and developmental stages [17,18,19,20], with total flavonoid content reaching 2360.9 ± 123.8 μg/g in mature fruit [18] and diosmin alone accounting for 1204.8 ± 1.4 μg/g in Guang Foshou [19], although absolute values differ appreciably between reports [17,21]. The position of Foshou within the genus *Citrus* is more informative than this internal variation. In *Citri reticulatae pericarpium*, the dried peel of *Citrus reticulata* and the most widely used citrus crude drug, the single flavanone glycoside hesperidin ranges from 27.5 to 86.3 mg/g across samples of different geographical origin [22], more than ten times the total flavonoid content of Foshou. The distinction, however, is compositional rather than quantitative. Whereas flavanone glycosides such as hesperidin, narirutin and naringin dominate the flavonoid pool of mandarin, pummelo and sweet orange peel, the most abundant flavonoid of Foshou is the flavone glycoside diosmin, which exceeds hesperidin at every developmental stage [18,19]. Guang Foshou therefore represents a flavone-dominant, diosmin-led chemotype that is uncommon within the genus and is not chemically interchangeable with other citrus medicinal materials. This chemotypic distinctiveness, combined with the pronounced tissue heterogeneity of the fruit, is what makes a tissue-resolved quality standard necessary and underlies the medicinal importance of Guang Foshou.

The ripening of Foshou is accompanied by a transition of the peel from green to golden yellow, a process that in citrus is under hormonal control. *Citrus* is a non-climacteric fruit that lacks the autocatalytic ethylene burst, yet peel degreening remains highly responsive to ethylene, which is exploited commercially in postharvest treatment [23]. Abscisic acid (ABA) is the other principal regulator, its rate-limiting step being catalysed by 9-cis-epoxycarotenoid dioxygenase, whose expression in the peel parallels ABA accumulation during maturation [24]. Both hormones converge on carotenoid metabolism: ethylene induces *CsCCD4b*, which generates the peel pigment β-citraurin, through a CsERF25–CsRP1 cascade [25], and the ABA-responsive module CsERF110–CsERF53 activates *CsPSY*, *CsZEP* and *CsNCED2* [26]. Notably, the same transcription-factor families also act on flavonoid metabolism, as AP2/ERF members activate the type IV chalcone isomerase *CitCHIL1* [27] and the R2R3-MYB factor *CsMYBF1* regulates flavonol biosynthesis [28].

Despite previous studies, the metabolomic profiles of flavonoids in Foshou remain incompletely characterized. A comprehensive understanding of the molecular mechanisms underlying fruit development and ripening would also provide essential information for the cultivation and production of Foshou. However, research on the developmental and ripening processes of Foshou remains limited. Differences in flavonoid composition and gene expression profiles between the zest (flavedo) and pith (albedo) of Foshou fruit have not yet been elucidated. Combined metabolomic and transcriptomic approaches have been successfully used to characterize biochemical pathways and metabolite accumulation in plants [29]. In this study, we similarly used a combined metabolomic and transcriptomic strategy to gain a more comprehensive understanding of the metabolic profiles of Guang Foshou. The metabolomes and transcriptomes of immature and mature Guang Foshou fruits were analyzed separately in the zest and pith tissues. Our results characterize the spatial and developmental dynamics of flavonoid accumulation and the associated regulatory processes in the zest and pith of Guang Foshou fruit, thus providing a clear scientific foundation for the molecular breeding of Foshou.

## 2. Materials and Methods

### 2.1. Plant Materials and Sample Collection

**Figure 1:**
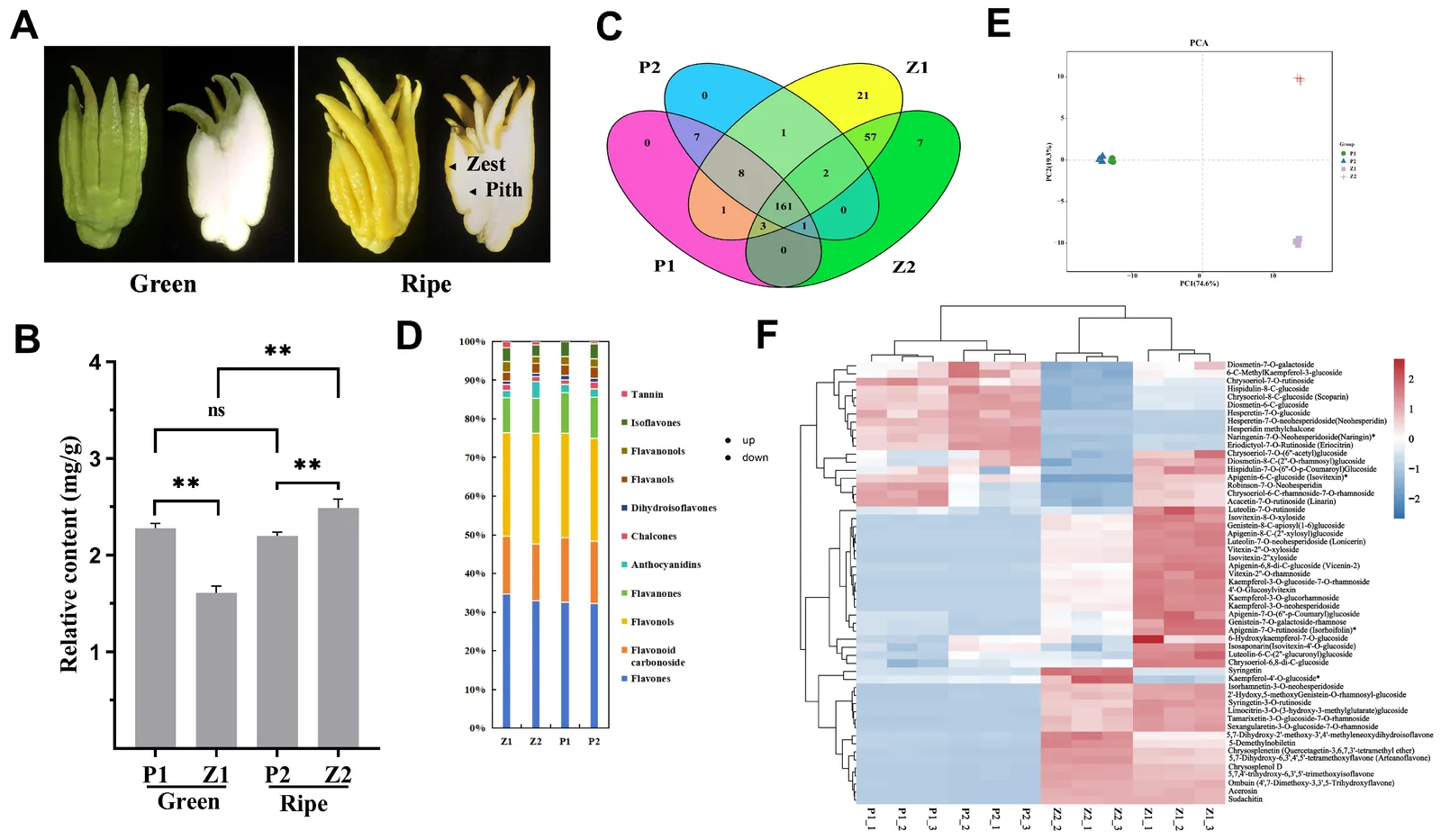
Morphological characteristics, flavonoid accumulation, and metabolomic profiling of Guang Foshou fruits at different developmental stages. Samples were designated as P1 (pith of green fruit), P2 (pith of ripe yellow fruit), Z1 (zest of green fruit), and Z2 (zest of ripe yellow fruit). (**A**) Representative images showing morphological changes in Guang Foshou fruit at the green and ripe stages. (**B**) Comparison of total flavonoid content across the four tissue groups, determined on fresh tissue and expressed on a fresh-weight basis (mean ± SD, *n* = 3). Asterisks indicate significant differences between the groups connected by brackets, assessed by two-tailed Student’s *t*-test: ** *p* < 0.01; ns, not significant. (**C**) Venn diagram showing the distribution of shared and group-restricted metabolites across the four tissue groups. Metabolites recorded as not detected in a group fell below the detection limit of the method and are not necessarily absent from the tissue. (**D**) Relative proportions of metabolite classes detected in the samples. (**E**) Principal component analysis (PCA) score plot showing metabolomic variation among the four sample groups. (**F**) Heatmap of 55 flavonoid metabolites with variable importance in projection (VIP) values ≥1.

Guang Foshou [*Citrus medica* ‘Fingered’] samples used in this study were obtained from the Lingnan Chinese Medicinal Materials Germplasm Resource Original Habitat Protection Base in Deqing County, Zhaoqing City, Guangdong Province, China (23° N, 111° E). The identity and authenticity of the specimens as genuine Southern medicinal materials were confirmed through collaborative taxonomic verification by the Deqing County Agricultural Technology Extension Center and the Deqing Zhongkai Agricultural Industry Technology Innovation Research Institute. Guang Foshou fruits were collected at two developmental stages, designated as the green-fruit and ripe-fruit stages. Green fruits were harvested on 23 September 2021, whereas ripe fruits were harvested on 20 October 2021. The fruit of *Citrus medica* ‘Fingered’ consists almost entirely of peel tissues, as the pulp is essentially undeveloped and juice vesicles are absent (Figure 1A). Two tissues were dissected: the thin outer pigmented layer bearing the oil glands, here termed the zest, which corresponds to the flavedo, and the thick inner white spongy tissue forming the bulk of the fruit, here termed the pith, which corresponds to the albedo. “Pith” is used throughout for this entire white spongy tissue, and not in the narrower sense of the central fruit axis. Zest and pith tissues were separately collected from three green fruits and three ripe fruits, as illustrated in Figure 1A. Samples were designated as Z1 and P1 for the zest and pith of green fruits, respectively, and Z2 and P2 for the zest and pith of ripe fruits, respectively. Three fruits were sampled from three different trees, and one fruit constituted one biological replicate; each group therefore comprised three independent biological replicates (*n* = 3). After collection, all tissue samples were rapidly frozen in liquid nitrogen and stored at −80 °C until further analysis.

### 2.2. Determination of Total Flavonoid Content (TFC)

Fresh zest and pith samples of Guang Foshou collected at two developmental stages were accurately weighed, with approximately 0.1 g of tissue used per analysis. Each sample was mixed with 1.5 mL of 60% ethanol and extracted by oscillating at 60 °C for 2 h. After extraction, the samples were centrifuged at 13,800× *g* and 25 °C for 10 min. The resulting supernatant was collected and adjusted to a final volume of 1.5 mL using 60% ethanol to prepare the test solution for total flavonoid quantification. Each biological replicate was assayed in three analytical replicates, the mean of which was taken as the value for that replicate. Total flavonoid content was measured using the NaNO_2_-Al(NO_3_)_3_-NaOH colorimetric method with a Plant Flavonoid Assay Kit according to the manufacturer’s protocol (spectrophotometric method; visible absorbance at 510 nm; Jining BiO, JN24727, Shanghai, China).

### 2.3. Metabolomic Analysis

Frozen zest and pith samples were dried using a vacuum freeze-dryer (Scientz-100F; Ningbo Scientz Biotechnology Co., Ltd. Ningbo, China). After complete drying, the samples were finely homogenized using a grinder (MM 400, Retsch, Haan, Germany) at 30 Hz for 1.5 min. Next, 100 mg of each powdered sample was extracted with 1.0 mL of 70% aqueous methanol containing 0.1 mg/L lidocaine as internal standard. The samples were incubated overnight at 4 °C and then centrifuged at 10,000× *g* for 10 min at 4 °C. The resulting supernatants were passed through a 0.22 μm Millipore membrane filter and collected for liquid chromatography-tandem mass spectrometry analysis. A quality-control sample was prepared by combining aliquots from all zest and pith samples. Samples were injected in two blocks, each preceded by a solvent blank and a quality-control injection and followed by a further pair, giving three blank and three quality-control injections across a sequence of 18; the full injection order is given in Table S1A. Widely targeted metabolomic profiling of Guang Foshou was performed on a Shim-pack UFLC system (CBM30A; Shimadzu Corporation, Kyoto, Japan) equipped with an ACQUITY UPLC HSS T3 C18 column (2.1 mm × 100 mm, 1.8 μm; Waters Corporation, Milford, MA, USA), coupled to a QTRAP 6500 triple quadrupole–linear ion trap mass spectrometer controlled by Analyst 1.6.1 software (AB Sciex, Framingham, MA, USA). The mobile phase consisted of 0.04% acetic acid in water as solvent A and 0.04% acetic acid in acetonitrile as solvent B. The flow rate was maintained at 0.4 mL/min. The chromatographic gradient was established as follows: the proportion of solvent B was initially set to 5% at 0 min and then increased linearly to 95% over 11 min. Solvent B was maintained at 95% from 11 to 12 min, reduced to 5% at 12.1 min, and maintained at 5% from 12.1 to 15 min. The column temperature was maintained at 40 °C, and the injection volume was 2 μL. The ion-source parameters were set as follows: ion-spray voltage, +5500/−4500 V; curtain gas pressure, 25 psi; ion-source gas I and gas II, 55 and 60 psi; ion-source temperature, 500 °C; and collision-activated dissociation, high. Mass spectra were acquired over the range *m*/*z* 50–1000 in both positive and negative ion modes. Instrument tuning and mass calibration were performed with 10 and 100 μmol L^−1^ polypropylene glycol solutions in triple-quadrupole and linear-ion-trap modes, respectively, and both quadrupoles were operated at unit mass resolution. Linear-ion-trap (LIT) and triple-quadrupole (QQQ) scans were both acquired: LIT scans were used to obtain product-ion spectra for metabolite identification, whereas quantification was performed by QQQ scans in multiple-reaction-monitoring (MRM) mode with nitrogen as the collision gas at 5 psi and with the declustering potential and collision energy optimized individually for each transition; the precursor (Q1) and product (Q3) ions of all compounds are listed in Table S1B. A separate set of MRM transitions was monitored in each retention-time period according to the metabolites eluting within that period. Metabolites were identified by matching precursor and product ions and fragmentation patterns against an in-house library of authentic standards (BioBioPha, BioBioPha Co., Ltd., Kunming, China; and Sigma-Aldrich, St. Louis, MO, USA) together with the MassBank, KNApSAcK, HMDB, MoTo DB and METLIN databases. Because isomeric flavonoid glycosides may share the same precursor-to-product transition, retention time was used as the criterion for distinguishing such compounds, by comparison with the retention times of the corresponding authentic standards in the provider’s library. Where two or more library isomers share a nominal transition, the annotation identifies the compound class and substitution pattern but is not resolved to the individual isomer; such entries are tentative at the isomer level and are indicated by the number of compounds sharing each nominal transition in Table S1B.

### 2.4. RNA-Seq Analysis

Total RNA was extracted from the collected samples using TRIzol reagent (Invitrogen, Carlsbad, CA, USA) as per the manufacturer’s instructions. RNA purity was assessed with a NanoPhotometer spectrophotometer, the OD_260_/_280_ values of the extracted RNA ranging from 1.8 to 2.1; RNA concentration was quantified with a Qubit 2.0 fluorometer, and RNA integrity was assessed with an Agilent 2100 Bioanalyzer (Agilent Technologies, Palo Alto, CA, USA) and by agarose gel electrophoresis. Messenger RNA was enriched using oligo(dT) beads. The enriched mRNA was fragmented into short fragments using fragmentation buffer and reverse-transcribed into cDNA using a reverse-transcription kit (NEB7530, New England Biolabs, Ipswich, MA, USA). The purified cDNA products were prepared for sequencing through end repair and tail addition. Sequencing was performed on an Illumina NovaSeq 6000 platform by Gene Denovo Biotechnology Co., Ltd. (Guangzhou, China). Raw reads were filtered with fastp v0.18.0 to remove reads containing adapters, reads with more than 10% unknown bases, reads consisting entirely of adenine and reads in which more than 50% of bases had a quality value of 20 or below; rRNA-derived reads were then removed by alignment to an rRNA database with Bowtie2 v2.2.8. Paired-end clean reads were mapped to the *Citrus medica* reference genome (Hzau v1) using HISAT2 v2.4 with the “--rna-strandness RF” option, and transcript assembly and abundance estimation were performed with StringTie v1.3.1. Transcript abundance was quantified as fragments per kilobase of transcript per million mapped reads (FPKM). Differentially expressed genes (DEGs) were identified using thresholds of an absolute log_2_ fold change of at least 1 and a false discovery rate < 0.05. The raw sequencing reads of the 12 libraries have been deposited in the Genome Sequence Archive of the National Genomics Data Center, China National Center for Bioinformation, under accession number CRA052375.

### 2.5. Data Processing and Statistical Analysis

All statistical analyses were based on biological replicates (*n* = 3 per group, each derived from a separate fruit on a separate tree). For the metabolomic and transcriptomic datasets each biological replicate was analyzed once, contributing a single observation to PCA, OPLS-DA and the *t*-tests; for the total flavonoid assay the three analytical replicates of each biological replicate were averaged beforehand. Metabolomic data were processed using Analyst v1.6.1. Peaks were retained at a signal-to-noise ratio greater than 10 after manual inspection, aligned across samples based on spectral pattern and retention time, and screened to remove redundant signals arising from isotopes, in-source fragmentation, K^+^, Na^+^ and NH_4_^+^ adducts and dimerization. Accurate *m*/*z* values were obtained for each Q1 ion. The total ion and extracted ion chromatograms of the quality-control samples were inspected to verify the overall reproducibility of the metabolite profiles. The coefficient of variation for each metabolite across the three quality-control injections was then calculated; the median was 7.2% and 92.9% of metabolites falling below 30% (Table S1B), and the median ratio of the last to the first quality-control injection was 0.97, indicating a systematic signal drift of 3.2% across the sequence. The area of each chromatographic peak was integrated and peak areas were unit-variance scaled. PCA was performed with the R package gmodels v2.18.1 and supervised partial least squares-discriminant analysis (PLS-DA) and orthogonal projections to latent structures-discriminant analysis (OPLS-DA) with the R package ropls. A PLS-DA model was built for all four groups, and an OPLS-DA model comprising one predictive and one orthogonal component was built for each pairwise comparison. Because R^2^Y approaches unity for any class assignment when the number of variables greatly exceeds the number of samples, model quality was judged by the cross-validated Q^2^ and by comparison with the permuted models rather than by R^2^Y. Because each comparison comprised only six samples, models were assessed by leave-one-out cross-validation and by exact permutation over all ten distinct partitions of the six samples, the smallest attainable *p*-value being 0.10; the four-group model was tested by 999 random permutations of the group labels. The multivariate models served for visualisation and VIP ranking, the significance of individual metabolites resting on the *t*-tests with Benjamini–Hochberg correction and on the two-way analysis of variance in Table S2. Variable Importance in Projection (VIP) scores were extracted from the predictive component of the OPLS-DA models and from the PLS-DA model. Metabolites were classified as differentially accumulated flavonoids (DAFs) only if they satisfied both of two criteria: a VIP score ≥ 1 in the corresponding OPLS-DA model and a Benjamini–Hochberg-adjusted *p*-value < 0.05 from a two-tailed Student’s *t*-test. Metabolites that reached an unadjusted *p* < 0.05 but not the adjusted threshold were not classified as DAFs and are not listed in Table S3. RNA-sequencing data were analyzed using DESeq2 software. Sequencing reads were aligned using HISAT2, and transcript abundance was quantified as FPKM mapped reads using StringTie. Genes meeting the criteria of an FDR < 0.05 and an absolute log_2_ fold change of at least 1 were classified as DEGs. Experimental groups were statistically compared using Student’s *t*-test based on the results of the analysis of variance. Differences with a *p*-value < 0.05 were considered statistically significant. The metabolite abundance table underlying all metabolomic analyses, comprising the integrated peak areas of the 269 metabolites in the 12 samples, has been deposited in the Open Archive for Miscellaneous Data of the National Genomics Data Center under accession number OMIX022528.

## 3. Results

### 3.1. Accumulation of TFC of Zest and Pith in Green and Yellow Fruit of Guang Foshou

To characterize differences in flavonoid accumulation associated with coloration and the formation of medicinal components in the zest and pith during the growth and maturation of Guang Foshou, the total flavonoid content of Z1, P1, Z2, and P2 was quantified. At the immature green-fruit stage, the total flavonoid content was significantly lower in the zest than in the pith, with values of 1.60 ± 0.08 mg/g in Z1 and 2.30 ± 0.05 mg/g in P1. In comparison, at the mature yellow-fruit stage, the total flavonoid content was significantly higher in the zest than in the pith, reaching 2.46 ± 0.09 mg/g in Z2 compared with 2.21 ± 0.05 mg/g in P2. Developmental changes showed a progressive accumulation of total flavonoids in the zest during fruit growth and maturation. By comparison, the total flavonoid content of the pith did not differ significantly between the two developmental stages (Figure 1B). These results indicate that maturation of Guang Foshou is associated with changes in the composition and distribution of flavonoid metabolites in the zest.

### 3.2. Identification of Differentially Accumulated Flavonoids (DAFs) of Zest and Pith in Green and Yellow Fruit of Guang Foshou

To characterize developmental and tissue-dependent changes in flavonoid metabolism, widely targeted metabolomic profiling was conducted on Z1, P1, Z2, and P2 samples. Using an independently established metabolite library and network database, 269 metabolites were detected, including 265 flavonoids and 4 tannins, along with their derivatives, distributed across 11 major metabolite classes (Table S1B). A total of 254, 231, 181, and 180 metabolites were detected in Z1, Z2, P1, and P2, respectively, and classified into 11 major groups (Figure S1A). Flavones, flavonols, flavonoid carbonosides, and flavanones accounted for 33.1%, 27.5%, 14.1%, and 8.9% of the identified metabolites, respectively. Tannins were detected in all sample groups except P1. Among the identified compounds, 161 metabolites were shared across all four tissue groups, whereas seven and 21 metabolites were detected only in Z2 and Z1, respectively (Figure 1C). These group-restricted detections should be interpreted with caution: the four groups differed in the total number of metabolites detected (254, 231, 181 and 180 in Z1, Z2, P1 and P2, respectively), and the 21 compounds detected only in Z1 were of low abundance, with a median peak area of 4.05 × 10^4^ compared with 8.25 × 10^4^ for the dataset as a whole. Among the 269 identified metabolites, flavones were the largest class, followed by flavonols, whereas dihydroisoflavones were the smallest class, containing only two metabolites (Figure 1D). Comparison of Z1 and Z2 showed no change in the number of dihydroisoflavones, which remained at two, or in the number of flavanols, which remained at six. Five additional anthocyanidins were detected in Z2, whereas reductions ranging from one to 12 metabolites were observed across eight metabolite classes. During the transition from P1 to P2, one additional metabolite was detected in each of the chalcone and tannin classes, whereas the number of flavones, flavonoid carbonosides, and flavonols each decreased by one metabolite (Figure 1D). These observations were consistent with the principal component analysis.

A two-way analysis of variance with tissue, developmental stage and their interaction as factors was performed for each of the 161 flavonoids detected in all four groups (Table S2). After Benjamini–Hochberg correction, tissue was significant for 150 metabolites, developmental stage for 114, and the tissue × stage interaction for 134. In the PCA score plot, the 12 samples formed four distinct clusters corresponding to the tissue types and developmental stages of Guang Foshou, with the 269 metabolites as variables (Figure 1E). Principal components 1 and 2 explained 74.6% and 19.3% of the total variance, respectively. The metabolic profiles of P1 and P2 were more similar than those of Z1 and Z2, indicating that the pith maintained relatively consistent metabolomic characteristics across development. The marked developmental differences between Z1 and Z2 indicate that flavonoid metabolism is closely associated with zest coloration and other maturation-related characteristics. The heatmap of 55 flavonoid metabolites with a variable importance in projection value of at least 1 in the four-group PLS-DA model is shown in Figure S2E. The magnitude of variation between Z1 and Z2 was greater than that observed between P1 and P2 (Figure 1F). Among these 55 metabolites, chrysoeriol-7-O-rutinoside, a flavone, exhibited the highest VIP value of 4.69 in the four-group PLS-DA model. Its abundance was substantially higher in the pith than in the zest, suggesting that chrysoeriol-7-O-rutinoside may serve as an important metabolite for distinguishing the two tissues of Guang Foshou. Four metabolites were detected only in the zest: kaempferol-3-O-neohesperidoside, kaempferol-3-O-glucoside-7-O-rhamnoside, kaempferol-3-O-glucorhamnoside, and luteolin-7-O-neohesperidoside, also known as lonicerin. The first three compounds were classified as flavonols, whereas luteolin-7-O-neohesperidoside was classified as a flavone. These four metabolites were detected in all six zest samples at mean peak areas of approximately 1.1 × 10^7^, about 130 times the median peak area of the dataset, while no signal was detected in any of the six pith samples. Although a level below the detection threshold cannot establish true absence, a difference of this magnitude indicates that their accumulation is strongly restricted to the zest. These results show that flavonoid accumulation in Guang Foshou is strongly associated with tissue type and developmental stage, with more differences in flavonoid composition occurring between immature green and mature yellow fruits. The four-group PLS-DA model gave a leave-one-out Q^2^ of 0.516, exceeding all but one of 999 permutations (*p* = 0.002). The pairwise OPLS-DA models gave leave-one-out Q^2^ values of 0.955 to 0.998, each exceeding all nine alternative partitions (−0.89 to −0.41), though with only ten partitions available the exact *p*-value cannot fall below 0.10. The score plots and the complete diagnostics of all five models are given in Figure S2, and the permutation results in Figure S3.

**Figure 2:**
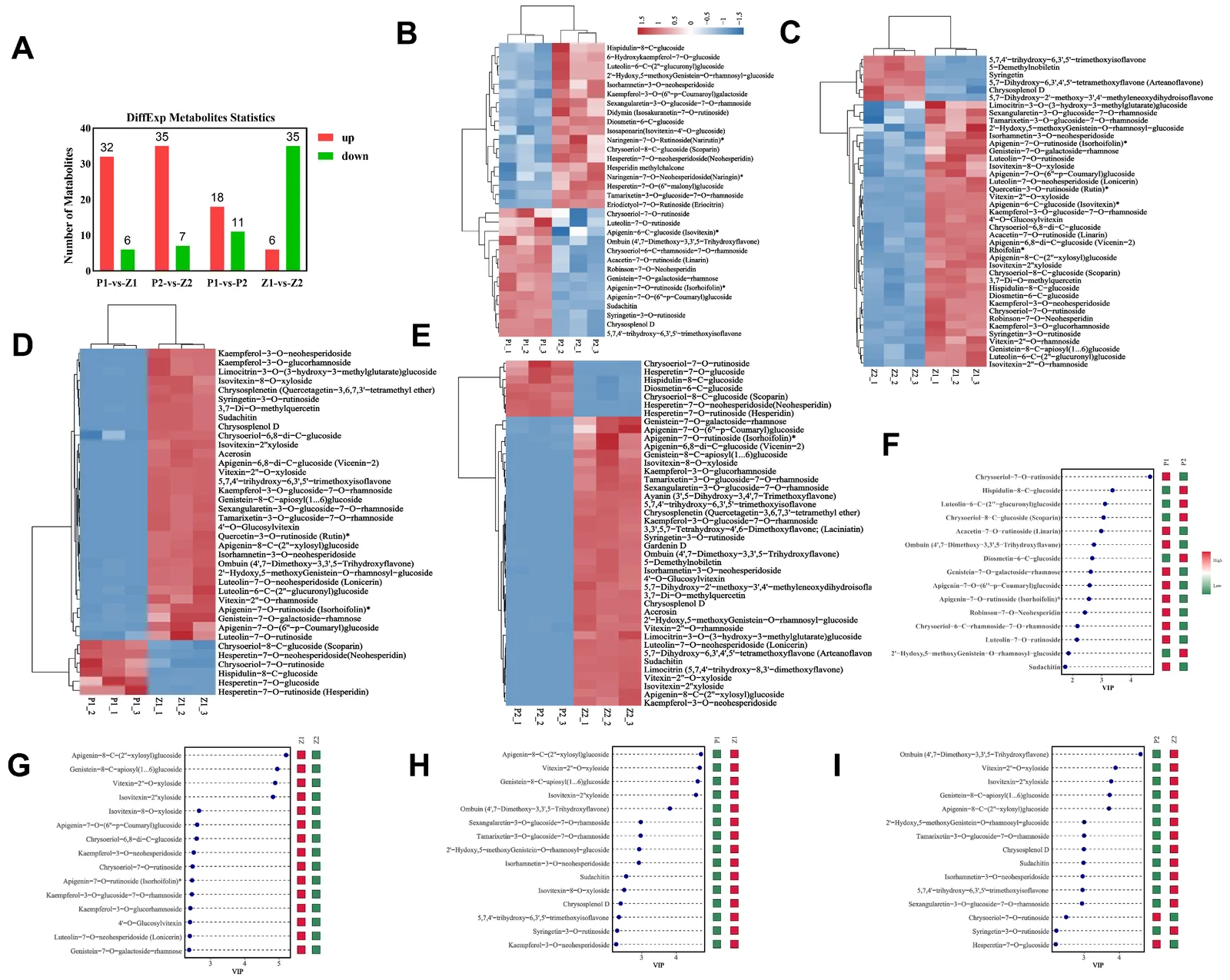
Comparative analysis of DAFs among four Guang Foshou tissue groups. (**A**) Number of upregulated and downregulated flavonoid metabolites identified in each pairwise comparison. (**B**–**E**) Heatmaps illustrating the accumulation of DAFs for the comparisons P1 vs. P2, Z1 vs. Z2, P1 vs. Z1, and P2 vs. Z2. An asterisk after a compound name indicates a metabolite whose isomers could not be resolved under the conditions used, so that the annotation is tentative at the isomer level. (**F**–**I**) VIP plots highlighting the key flavonoid metabolites that contribute most to the metabolic differences observed in each comparison group.

To further detect patterns and differences in flavonoid accumulation among the four sample groups, an OPLS-DA model was developed. DAFs were identified using thresholds of VIP ≥1 and a Benjamini–Hochberg-adjusted *p* ≤ 0.05. A total of 29 DAFs were identified between P1 and P2, 41 between Z1 and Z2, 38 between P1 and Z1, and 42 between P2 and Z2. In all developmental stages, six metabolites were upregulated, and 35 were downregulated between Z1 and Z2. Between P1 and P2, 18 metabolites were upregulated, and 11 were downregulated. Across tissue comparisons, 32 metabolites were upregulated, and six were downregulated between P1 and Z1, whereas 35 were upregulated and seven were downregulated between P2 and Z2 (Figure 2A–E; Table S3). In the P1-vs-P2 comparison, hispidulin-8-C-glucoside, luteolin-6-C-(2″-glucuronyl)-glucoside and chrysoeriol-8-C-glucoside, also known as scoparin, displayed VIP values of 3.37, 3.11 and 3.06, respectively, and all three were less abundant in P1 than in P2. Chrysoeriol-7-O-rutinoside showed the highest VIP value in this comparison (4.65) and was more abundant in P1 than in P2, but the difference did not remain significant after correction for multiple testing (adjusted *p* = 0.144) and this metabolite is therefore not considered further as a stage-discriminating compound of the pith. Its abundance did, however, differ strongly between the two tissues at both stages (adjusted *p* = 1.3 × 10^−3^ for P1 vs. Z1 and 1.4 × 10^−3^ for P2 vs. Z2). Chrysoeriol-6,8-di-C-glucoside was the most abundant metabolite in both P1 and P2 and showed equivalent abundance in the two groups, followed by chrysoeriol-7-O-rutinoside. In the Z1-vs-Z2 comparison, apigenin-8-C-(2″-xylosyl)-glucoside, genistein-8-C-apiosyl-(1-6)-glucoside, vitexin-2″-O-xyloside, and isovitexin-2″-xyloside exhibited VIP values of 5.20, 4.95, 4.89, and 4.82, respectively. All four metabolites were less abundant in Z2 than in Z1. Among these compounds, vitexin-2″-O-xyloside showed the highest abundance in Z1, followed by genistein-8-C-apiosyl-(1-6)-glucoside, and both metabolites were present at approximately twice the abundance observed in Z2. Ombuin, or 4′,7-dimethoxy-3,3′,5-trihydroxyflavone, was the most abundant metabolite detected in Z2 and showed similar abundance in Z1. In the P1-vs-Z1 comparison, apigenin-8-C-(2″-xylosyl)-glucoside, vitexin-2″-O-xyloside, genistein-8-C-apiosyl-(1-6)-glucoside, and isovitexin-2″-xyloside showed VIP values of 4.72, 4.68, 4.62, and 4.57, respectively. All four metabolites were less abundant in P1 than in Z1. In the P2-vs-Z2 comparison, ombuin, vitexin-2″-O-xyloside, isovitexin-2″-xyloside, and genistein-8-C-apiosyl-(1-6)-glucoside exhibited VIP values of 4.60, 3.89, 3.77, and 3.72, respectively. Each of these metabolites was less abundant in P2 than in Z2 (Figure 2F–I; Table S3). These results indicate that flavonoid carbonosides represent the main metabolite class distinguishing the pith and zest of Guang Foshou across developmental stages.

**Figure 3:**
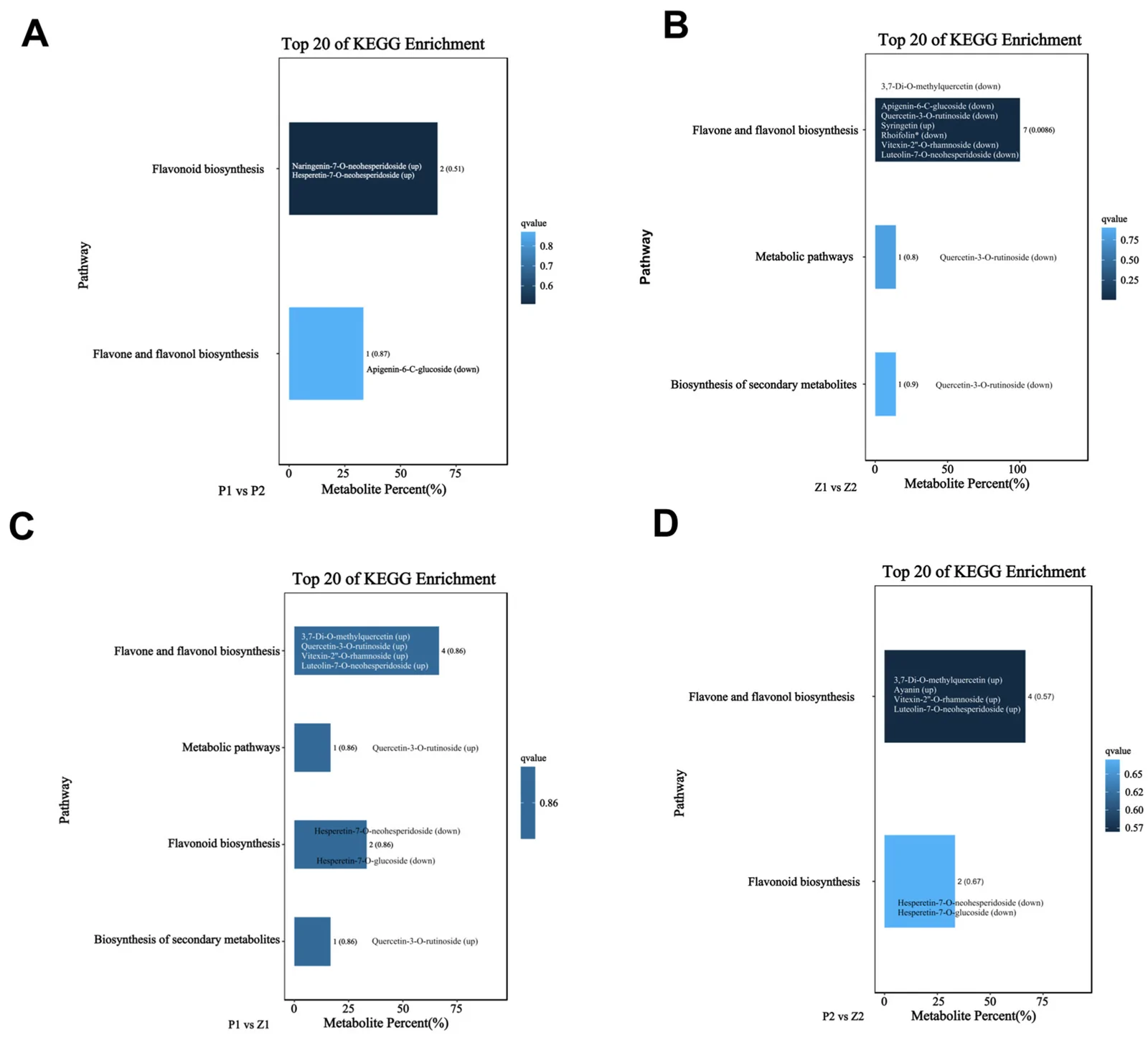
Kyoto Encyclopedia of Genes and Genomes (KEGG) flavonoid pathways between four different types of tissues. (**A**). KEGG pathway of P1 vs. P2. (**B**). KEGG pathway of Z1 vs. Z2. (**C**). KEGG pathway of P1 vs. Z1. (**D**). KEGG pathway of P2 vs. Z2. P1, pith of green fruit; P2, pith of ripe yellow fruit; Z1, zest of green fruit; and Z2, zest of ripe yellow fruit. An asterisk after a compound name indicates a metabolite whose isomers could not be resolved under the conditions used, so that the annotation is tentative at the isomer level.

To identify the biochemical pathways associated with the DAFs, enrichment analysis was performed using the Kyoto Encyclopedia of Genes and Genomes (KEGG) database. Four metabolic pathways were associated with the zest and pith of green and yellow Guang Foshou fruits, and the pathway differences between the tissue comparisons are presented in Figure 3A–D. The main DAFs associated with the flavonoid biosynthesis pathway were hesperetin-7-O-neohesperidoside and hesperetin-7-O-glucoside. Both metabolites were upregulated between P1 and P2 but downregulated in the P1-vs-Z1 and P2-vs-Z2 comparisons.

### 3.3. Transcriptomic Analysis During the Development and Ripening of Guang Foshou

**Figure 4:**
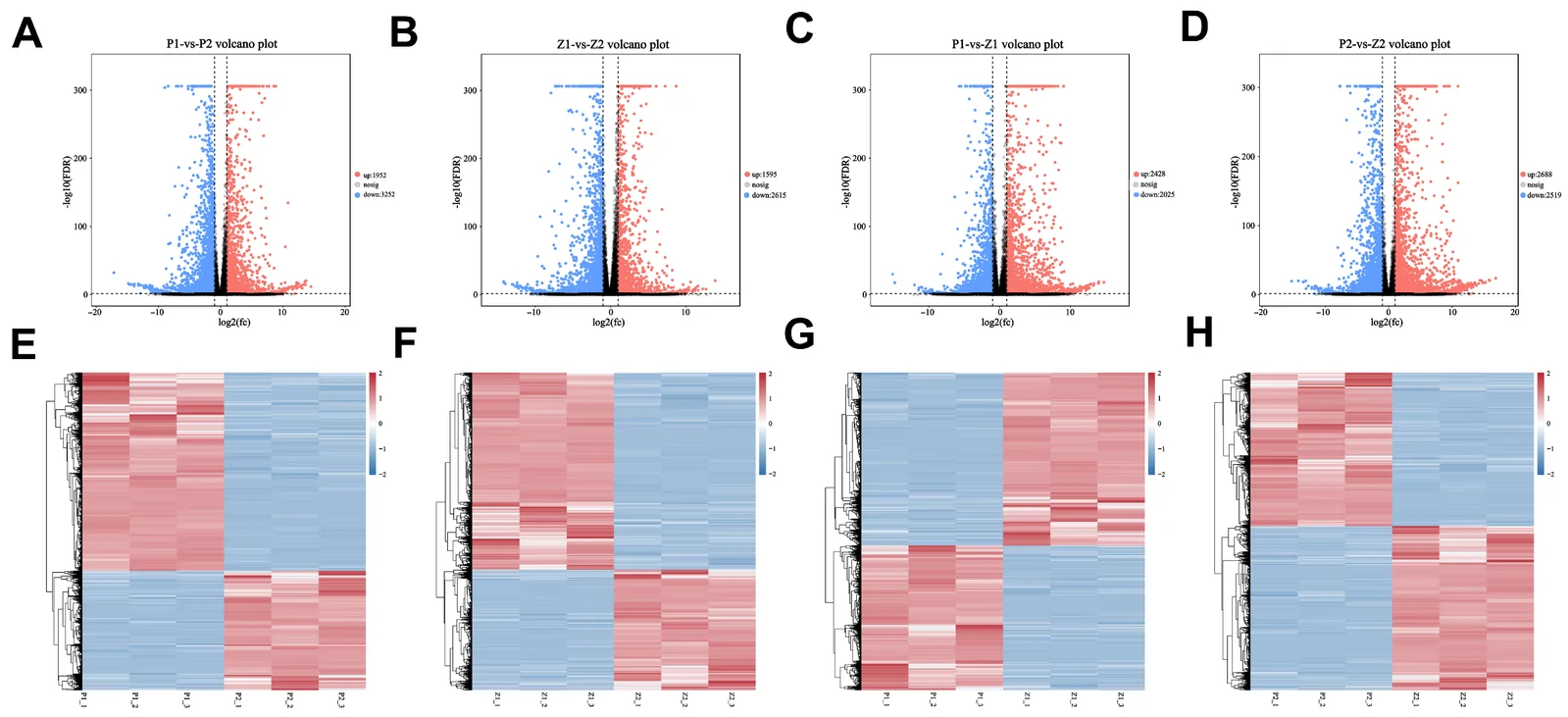
Volcano plots and heat maps to analyze differentially expressed gene (DEG) patterns of P1, P2, Z1, and Z2. (**A**–**D**). Volcano plots showing the DEGs across four tissue-group comparisons: P1 vs. P2, Z1 vs. Z2, P1 vs. Z1, P2 vs. Z2; (**E**–**H**). Heatmaps showing the DEG patterns between four tissue comparison groups. P1, pith of green fruit, P2, pith of ripe yellow fruit, Z1, zest of green fruit, and Z2, zest of ripe yellow fruit.

As systematic transcriptomic characterization of Guang Foshou remains limited, transcriptome sequencing was conducted to elucidate the biosynthetic mechanisms underlying the production of bioactive flavonoid metabolites. Transcriptomic profiles of the pith and zest were examined at two developmental stages to characterize changes in gene expression during fruit growth and maturation. Twelve RNA-sequencing libraries, three for each tissue-stage group, were sequenced as 150 bp paired-end reads on the Illumina NovaSeq 6000 platform, yielding 570.0 million clean reads in total (42.2 to 56.1 million per library, 85.2 Gb), with Q30 above 92.0% in every library and residual rRNA below 0.5%. Clean reads were mapped to the *Citrus medica* reference genome (Hzau v1) using HISAT2, with total mapping rates of 96.2% to 97.8% per library, of which 86.5% to 88.4% mapped to unique positions. Of the 33,952 genes in the reference annotation, 23,771 were expressed (FPKM > 0) in at least one library, with between 19,332 and 20,342 genes expressed per library. The per-sample sequencing, filtering and alignment statistics are given in Table S4. Read count data were analyzed for differential gene expression using DESeq2. Significantly DEGs were identified using FDR <0.05 and |log_2_FC| >1. The distributions of DEGs across the four comparison groups are shown in Figure 4A–D. For the tissue comparisons, 4453 DEGs were identified between P1 and Z1, with 2428 upregulated and 2025 downregulated genes. In the P2-vs-Z2 comparison, 5207 DEGs were detected, with 2688 upregulated and 2519 downregulated genes. These comparisons showed a modest predominance of upregulated genes in the pith. For developmental comparisons, 5208 DEGs were detected between P1 and P2, including 1956 upregulated and 3252 downregulated genes. Between Z1 and Z2, 4210 DEGs were identified, comprising 1595 upregulated and 2615 downregulated genes. The predominance of downregulated genes in both comparisons indicates extensive transcriptional suppression during maturation of the yellow fruit. Heatmap analysis revealed distinct gene expression profiles between the pith and zest and a close association with developmental stage (Figure 4E–H). These observations indicate that gene expression during Guang Foshou development is both tissue- and stage-dependent. PCA of the transcriptomic data showed that P1 and Z1 clustered closely together (Figure S1B), indicating that the pith and zest showed similar transcriptional profiles during the early stage of fruit development. In comparison, significant transcriptomic differences were observed between the pith and zest at the mature stage, as shown by the separation of P2 and Z2 in Figure S1B. Gene Ontology terms were grouped into three major categories: biological processes (BP), cellular components (CC), and molecular functions (MF) (Figure S4). KEGG analysis identified 133 enriched pathways in P1 vs. P2, 130 in Z1 vs. Z2, 125 in P1 vs. Z1, and 131 in P2-vs-Z2. In the P1-vs-P2 comparison, the four most highly enriched pathways were metabolic pathways, biosynthesis of secondary metabolites, ribosome, and carbon metabolism (Figure S4). In the Z1-vs-Z2 and P1-vs-Z1 comparisons, the most enriched pathways included phenylpropanoid biosynthesis, secondary metabolite biosynthesis, metabolic pathways, and plant hormone signal transduction. In the P2-vs-Z2 comparison, the most strongly enriched pathways were phenylpropanoid biosynthesis, secondary metabolite biosynthesis, metabolic pathways, carbon metabolism, and amino acid biosynthesis. These results indicate that general metabolic processes and secondary/specialized -metabolite biosynthesis play essential roles in the development of Guang Foshou fruit.

### 3.4. Phytohormone-Related Pathways Associated with the Green-to-Yellow Transition

**Figure 5:**
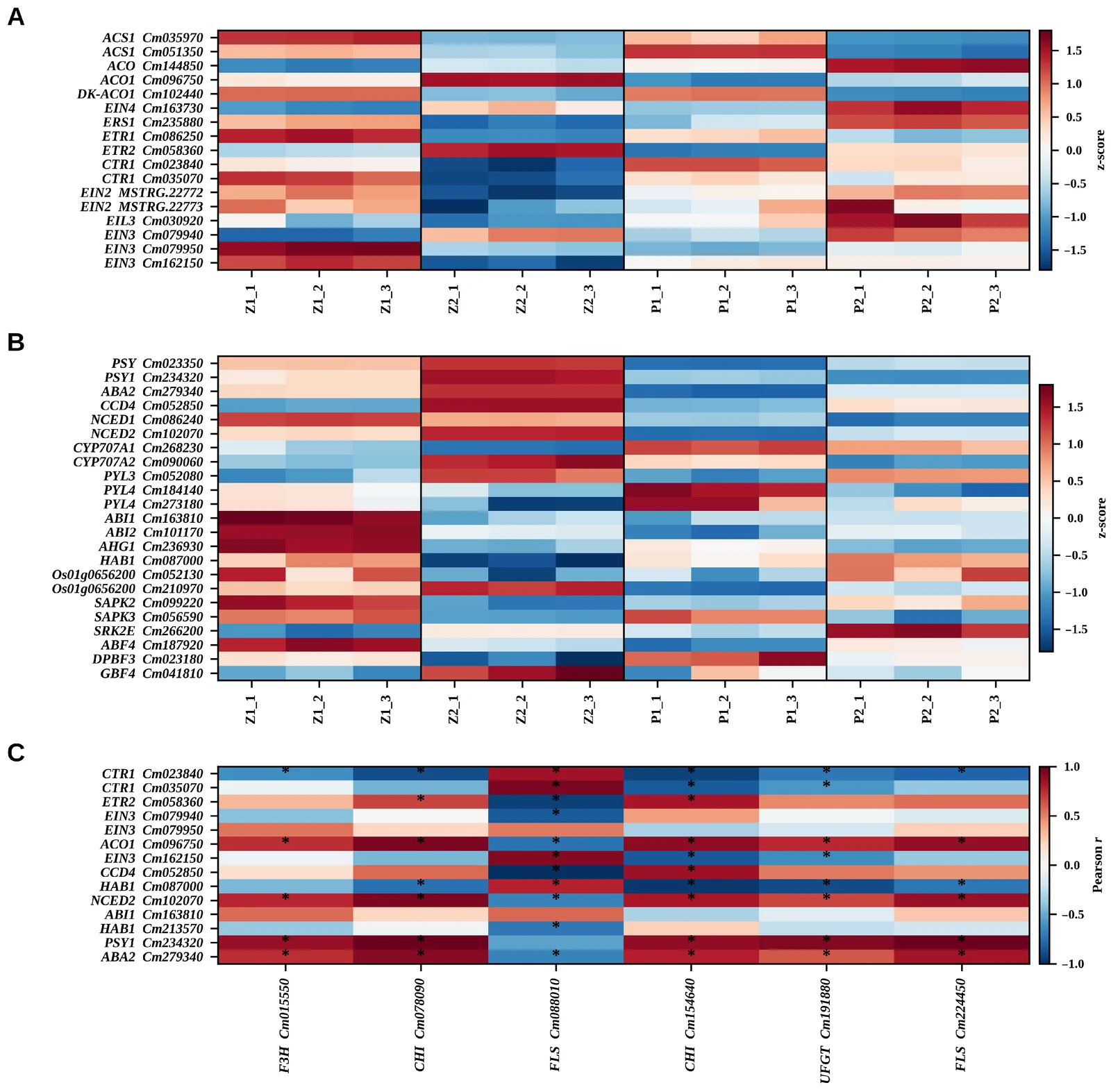
Expression of phytohormone-related genes and their association with flavonoid structural genes in the zest and pith of Guang Foshou. (**A**) Heat map of ethylene biosynthesis (*ACS*, *ACO*) and signalling (*ETR/ERS*, *CTR1*, *EIN2*, *EIN3/EIL*) genes across the four tissue–stage groups, shown as log_2_(FPKM + 1) with row-wise z-score scaling; the colour bar indicates the z-score. (**B**) Heat map of ABA biosynthesis (*PSY*, *ZEP*, *NCED*, *CCD4*), catabolism (*CYP707A*) and signalling (*PYL*, *PP2C*, *SnRK2*, *ABF)* genes, scaled as in (**A**). (**C**) Pearson correlation matrix between hormone-related genes and the flavonoid structural genes *CHS*, *CHI*, *FLS*, *CYP75B2*, *FGT* and *UFGT*, calculated on log_2_(FPKM + 1) values across all 12 samples; Asterisks indicate significant differences between the groups connected by brackets, assessed by two-tailed Student’s *t*-test: * *p* < 0.05. Z1, zest of green fruit; Z2, zest of ripe yellow fruit; P1, pith of green fruit; P2, pith of ripe yellow fruit.

Because the green-to-yellow transition of citrus zest is known to be hormonally regulated, the transcriptomic data were further examined for phytohormone biosynthesis and signalling components. KEGG enrichment analysis showed that “plant hormone signal transduction” (ko04075) was significantly “Carotenoid biosynthesis” (ko00906), which supplies the precursors of abscisic acid (ABA), was enriched in three of the four comparisons (20 genes, q = 0.0015 for Z1 vs. Z2 17 genes, q = 0.014 for P1 vs. Z1 20 genes, and q = 0.0034 for P2 vs. Z2). Pathways related to brassinosteroid biosynthesis and α-linolenic acid metabolism, the latter being the precursor route of jasmonic acid, were also enriched in the zest comparisons, indicating that the transition involves multiple hormonal inputs rather than a single regulator. Within the ethylene pathway, the expression of two 1-aminocyclopropane-1-carboxylate oxidase genes increased significantly in the zest during ripening (*ACO1*, Cm096750; *ACO*, Cm144850; both *p* < 0.001), whereas the ACC synthase genes *ACS1* (Cm035970, Cm051350) were downregulated. Among the receptor genes, *ETR2* (Cm058360) and EIN4 (Cm163730) were upregulated, while *ETR1* and *ERS1* were downregulated. Notably, both copies of the negative regulator *CTR1* (Cm035070, Cm023840) were significantly downregulated in the zest, and the downstream transcription factor gene *EIN3* (Cm079940) was upregulated. This combination—increased *ACO* transcript levels and receptor expression together with reduced *CTR1* expression—is consistent with activation of ethylene signalling in the zest, while the concurrent downregulation of ACS indicates that the tissue does not enter the autocatalytic ethylene burst characteristic of climacteric fruit (Figure 5A). The ABA pathway showed even stronger changes. The 9-cis-epoxycarotenoid dioxygenase gene *NCED2* (Cm102070) was the most highly expressed hormone-related gene in the dataset and increased from 1117.6 to 4390.9 FPKM during zest ripening, accompanied by marked upregulation of *CCD4* and of the zeaxanthin epoxidase gene *ZEP*. Upstream, both phytoene synthase genes were upregulated (*PSY1* and *PSY*), indicating enhanced precursor supply. In the signalling branch, four clade-A protein phosphatase 2C genes acting as negative regulators were coordinately downregulated (*AHG1*, *HAB1*, *ABI1*, −1.04; *ABI2*, all *p* < 0.001), consistent with de-repression of ABA signalling in the zest (Figure 5B). To relate these regulators to flavonoid metabolism, the expression of hormone-related genes was correlated with that of the flavonoid structural genes across all 12 samples. *ACO1* was positively correlated with *CHI3* (Cm078090) and with *FLS* (Cm088010), and *NCED2* was positively correlated with *CHI3* and *FLS*. The strongest association in the dataset was the negative correlation between *CCD4* and *FLS*, and *PSY1* was positively correlated with *CHI3*, *FLS* and *FGT* (Figure 5C). These co-expression relationships suggest that ethylene- and ABA-related components are transcriptionally coordinated with the flavonoid pathway during the green-to-yellow transition, although with four sample groups the analysis establishes association rather than causality.

### 3.5. Flavonoid Pathways and Their Related Genes

Combined transcriptomic and metabolomic analyses identified the following co-enriched pathways: metabolic pathways, secondary metabolite biosynthesis, flavonoid biosynthesis, flavone and flavonol biosynthesis, isoflavonoid biosynthesis, and anthocyanin biosynthesis. These results show that substantial metabolic reprogramming occurs during the development and ripening of Guang Foshou, with these alterations predominantly involving secondary metabolism. Flavonoid biosynthesis is a key metabolic network linking upstream phenylpropanoid metabolism to multiple downstream branches, including flavone and flavonol biosynthesis, isoflavonoid biosynthesis, and anthocyanin biosynthesis. Accordingly, gene expression patterns associated with these four pathways were examined in the zest and pith to identify key genes and metabolic branches contributing to the contrasting flavonoid profiles of the two tissues.

**Figure 6:**
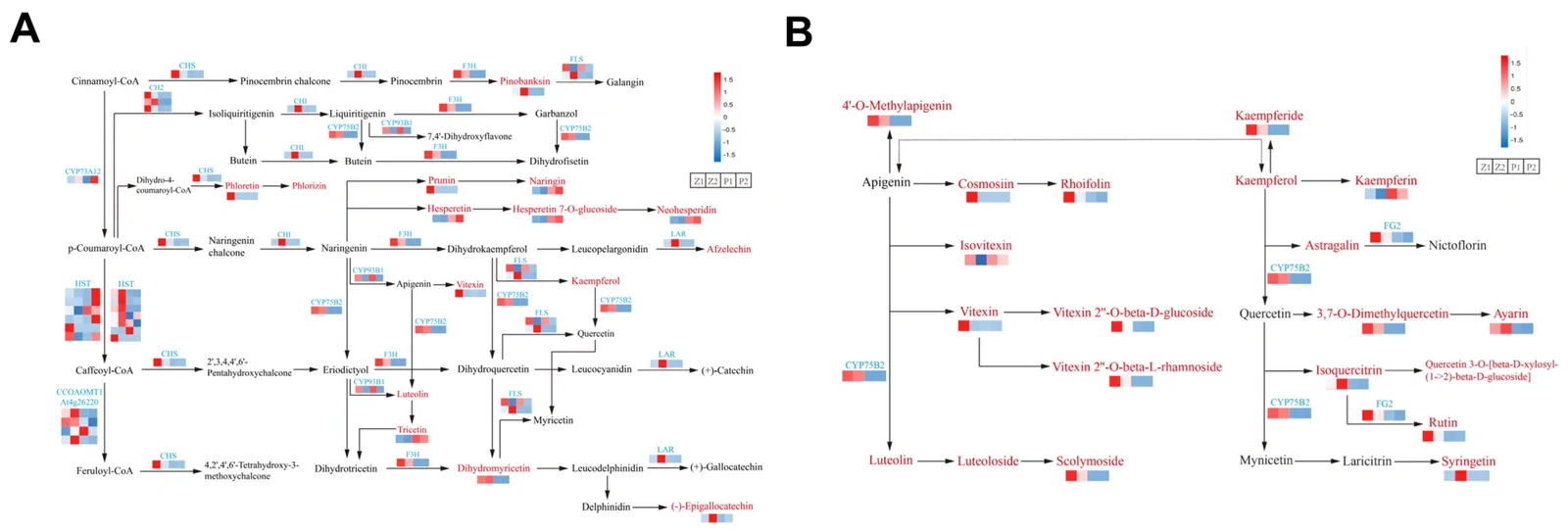
Differential gene expression map of flavonoid biosynthesis in Guang Foshou. (**A**) Pathway map showing flavonoid products derived from cinnamoyl-CoA. (**B**) Pathways of flavone and flavonoid biosynthesis showing the contrasting patterns in zest and pith. P1, pith of green fruit; P2, pith of ripe yellow fruit; Z1, zest of green fruit; and Z2, zest of ripe yellow fruit.

In the flavonoid biosynthesis pathway, the galangin branch was enriched in the zest and involved *CHS*, *F3H*, and *FLS* (Figure 6A). The main intermediate p-coumaroyl-CoA, produced through *CYP73A12*, contributed to the formation of dihydrofisetin, phloretin/phlorizin, and 4,2′,4′,6′-tetrahydroxy-3-methoxychalcone, with the expression of the corresponding biosynthetic genes shown in Figure 6. These compounds were enriched in the zest of green fruits. Naringenin, a crucial intermediate synthesized via *CHS* and *CHI*, served as a precursor to prunin and naringin. In the pith, prunin, the upstream metabolite, and naringin, the downstream metabolite, showed an inverse relationship during fruit development, indicating a progressive conversion of prunin to naringin. Naringenin also contributed to hesperidin biosynthesis. All hesperidin-associated metabolites were significantly enriched in the pith and accumulated progressively as fruit matured. Dihydrokaempferol, afzelechin, kaempferol, apigenin, vitexin, luteolin and tricetin, which lie downstream of naringenin in the pathway, were all detected, and their abundances are shown in Figure 6A. Dihydromyricetin and epigallocatechin, the products of tricetin in the pathway, were both more abundant in the zest and accumulated significantly in the zest of mature fruits.

In the flavone and flavonol biosynthesis pathway, cosmosiin and rhoifolin showed similar abundance across all tissues and developmental stages, indicating a stable relationship between these upstream and downstream metabolites (Figure 6B). Vitexin, located upstream of vitexin-2″-O-rhamnoside and vitexin-2″-O-glucoside, reached its highest abundance in the green zest (Z1). Kaempferol was another metabolite involved in the formation of kaempferide, which was predominantly enriched in the zest, and of kaempferin, which was enriched in the pith. The abundance of 3,7-O-dimethylquercetin declined and that of ayanin rose during zest development, a pattern consistent with conversion of the former to the latter, with ayanin reaching its highest abundance in yellow fruits. *FG2*, which encodes the enzyme catalysing the conversion of isoquercitrin to rutin, was most highly expressed in the green zest (Z1), where the rutin-to-isoquercitrin abundance ratio was also highest. *CYP75B2* was highly expressed in the zest, supporting its involvement in the conversion of apigenin to luteolin and of kaempferol to quercetin.

**Figure 7:**
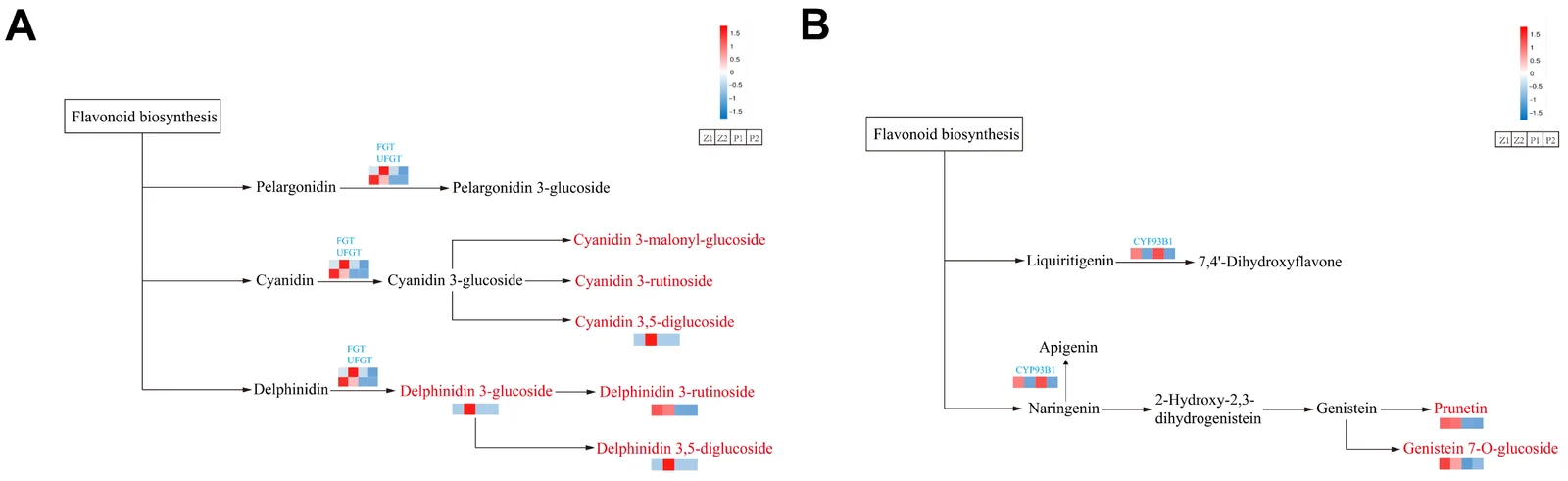
Flavonoid biosynthesis gene pathway maps showing the anthocyanin and isoflavonoid branches in Guang Foshou. (**A**) Isoflavonoid biosynthetic pathway. (**B**) Anthocyanin biosynthetic pathway. P1, pith of green fruit; P2, pith of ripe yellow fruit; Z1, zest of green fruit; and Z2, zest of ripe yellow fruit.

In the isoflavonoid biosynthesis pathway, prunetin and genistein-7-O-glucoside were both derived from genistein and were more abundant in the zest of Guang Foshou (Figure 7A). *CYP93B1*, which encodes the flavone synthase II catalysing the conversion of naringenin to apigenin and of liquiritigenin to 7,4′-dihydroxyflavone, was more highly expressed in green fruits. In the anthocyanin biosynthesis pathway, cyanidin-3-malonyl-glucoside, cyanidin-3-O-rutinoside, and cyanidin-3,5-O-diglucoside were derived from cyanidin-3-glucoside (Figure 7B). Cyanidin-3,5-O-diglucoside accumulated primarily in the zest of mature fruit. Delphinidin-3-glucoside served as the precursor to delphinidin-3-rutinoside and delphinidin-3,5-diglucoside. All three compounds accumulated to higher levels in the zest, with delphinidin-3-rutinoside reaching its maximum abundance in the zest of green fruits. The key DEGs, *FGT* and *UFGT*, regulate the conversion of pelargonidin, cyanidin, and delphinidin into their downstream derivatives. *UFGT* is highly expressed in the zest of green fruits, whereas *FGT* shows significant expression in the zest of mature fruits, suggesting synergistic roles in anthocyanin conversion during fruit development. These results highlight that the accumulation of flavonoids, isoflavonoids, and anthocyanins in Guang Foshou differed with tissue type and developmental stage. The expression of *CYP93B1*, *FGT* and *UFGT* covaried with the abundance of their respective pathway products, identifying them as candidate determinants of flavonoid composition. These assignments follow the reactions annotated in the KEGG flavonoid pathways and are supported by the covariation of transcript and metabolite abundance; they were not verified by enzyme-activity assays or isotopic flux measurements, and the directionality of individual steps is therefore inferred from the pathway annotation rather than measured in this study.

## 4. Discussion

### 4.1. Enhanced Medicinal Value of Yellow Fruit and the Scientific Basis for the “Yellow Zest with White Pith” Quality Criterion

During the maturation of Guang Foshou from the green to the yellow fruit stage, the total flavonoid content of the zest increased significantly from 1.60 ± 0.08 mg/g to 2.46 ± 0.09 mg/g, whereas the total flavonoid content of the pith remained relatively stable at 2.20–2.30 mg/g. These results show a distinct difference in flavonoid accumulation between the zest and pith. The zest accounted for the majority of the increase in total flavonoids, while the pith maintained a comparatively constant basal concentration. At the metabolite level, the yellow zest selectively accumulated polymethoxylated flavones (PMFs), including nobiletin, tangeretin, 5-demethylnobiletin, and the methylated flavonol syringetin. Zhao et al. (2020) reviewed the health-related effects of citrus flavonoids and emphasized the prominent anti-inflammatory, anticancer, and neuroprotective activities of PMFs [30]. Consistent with these observations, Guo et al. showed that 5-demethylnobiletin and its metabolites suppressed the expression of inducible nitric oxide synthase and cyclooxygenase-2 in lipopolysaccharide-induced macrophages and showed stronger anti-inflammatory activity than the parent compound [31]. In comparison, the yellow pith accumulated high concentrations of flavanone glycosides (hesperidin, neohesperidin, naringin, and eriocitrin). Hesperidin possesses several beneficial biological properties, including hypolipidemic, anti-inflammatory, and antiviral activities. However, its biological availability is limited by poor intestinal absorption and low aqueous solubility. Physical, chemical, and biological modification strategies may therefore be used to substantially improve its solubility and bioavailability [32]. Accordingly, the traditional quality criterion of “yellow zest with white pith” has a clear phytochemical basis. The zest accumulates highly bioactive PMFs, whereas the pith serves as a reservoir of flavor-associated and cardioprotective flavanone glycosides. In traditional Chinese medicine (TCM), the quality-marker concept, also referred to as the Q-marker, emphasizes that quality assessment should target components that are specific to particular tissues or medicinal parts [33]. From a medicinal perspective, these tissue- and stage-dependent differences in flavonoid composition are relevant to the selection of raw material, although they do not by themselves establish a difference in therapeutic efficacy. Our analysis was confined to flavonoids, whereas the medicinal properties of Foshou also involve coumarins, essential oils and polysaccharides that were not measured here; moreover, ripening did not increase flavonoid levels uniformly, since 35 flavonoid glycosides declined significantly in the zest while methoxylated products accumulated, and in the pith total flavonoid content did not change significantly. Bioavailability further complicates any inference from content to effect: polymethoxylated flavones are highly bioactive in vitro but poorly absorbed and sparingly water-soluble, and the pharmacological activity of a herbal material reflects the combined and potentially synergistic action of its constituents rather than the content of any single class. We therefore interpret our results as a compositional rather than a pharmacological distinction: the mature yellow fruit differs from the green fruit in the profile of flavonoids it accumulates, which provides a chemical basis for stage-dependent quality control, while comparative pharmacological or clinical evaluation would be required to determine whether this translates into differences in therapeutic effect.

### 4.2. Flavonoid Metabolic Patterns Accompanying Color Transition and Identification of Maturity Markers

The transition of Guang Foshou zest from green to yellow was closely associated with extensive reprogramming of flavonoid metabolism. During zest maturation, 35 flavonoid glycosides were significantly downregulated, with log_2_ fold-change values ranging from −1.0 to −4.0. These compounds consisted predominantly of C-glycosylflavones, including apigenin-8-C-(2″-xylosyl)-glucoside and vitexin-2″-O-xyloside, together with O-glycosylated flavones and flavonols, including luteolin-7-O-rutinoside and kaempferol-3-O-neohesperidoside. This metabolic profile is similar to the “two-phase flavonoid formation” pattern observed in strawberries, in which glycosides accumulated during the early developmental stage provide ultraviolet absorption and antioxidant protection to young tissues [34]. Early flavonol accumulation has also been reported in peaches, where it occurs alongside lignin biosynthesis during stone-cell formation [35]. These conserved patterns across plant species suggest that C-glycosides and O-glycosides function as a form of “chemical armor” during the immature stage of fruit development. The flavonoid methoxylation pathway was selectively activated in the zest, resulting in the upregulation of PMFs such as 5-demethylnobiletin, arteanoflavone, and chrysosplenol D. This observation suggests that the metabolic shift toward methoxylation during fruit maturation and the subsequent effects of aging may share a similar chemical basis, as both processes are associated with a shift from hydrophilic glycosides towards lipophilic and highly bioactive methoxylated end products. Based on these dynamic metabolic patterns, three groups of developmental-stage-dependent chemical markers are proposed. Green fruit is characterized by C-glycosylflavones, including isovitexin-2″-xyloside and vicenin-2; yellow zest is characterized by PMFs, including nobiletin, tangeretin, and the methylated flavonol syringetin; and yellow pith is identified by flavanone glycosides, including neohesperidin, naringin, and hesperidin. This study complements previous findings by identifying nonvolatile flavonoid markers. The combined assessment of nonvolatile and volatile markers may provide a more comprehensive basis for determining harvest timing. Transcriptomic analysis further supported the observed metabolic patterns. Key genes involved in flavonoid biosynthesis, including *CHS*, *CHI*, *FLS*, *CYP75B2*, and *FGT*, showed tissue- and developmental-stage-dependent expression patterns that were highly consistent with the respective metabolite profiles. Multiple DEGs associated with terpene biosynthesis have also been identified in finger citron, indicating that transcriptional regulation is essential for the tissue-dependent distribution of secondary metabolites [36]. In addition to biosynthetic genes, metabolite transporters, including *CmABCB19* and *CmABCC10* in *Citrus medica*, have been analyzed, supporting the role of ATP-binding cassette transporters in the vacuolar transport of flavonoids [37]. These transporters may regulate the distribution of glycosides between the zest and pith, providing molecular support for the “source-sink” hypothesis. This study focused on genes associated with four major metabolic pathways. However, numerous genes are likely to participate in the regulation of Guang Foshou development. Therefore, further characterization of these functionally important genes will be crucial to obtaining a comprehensive understanding of the regulatory network underlying Guang Foshou fruit development.

The green-to-yellow transition of citrus zest is hormonally controlled, and our data place the flavonoid changes described above in this context. *Citrus* is non-climacteric, yet its zest remains highly ethylene-responsive, and a system II-like pathway of ethylene production operates in non-climacteric citrus fruit [23]. Our results fit this physiology: in the ripening zest, two ACC oxidase genes and the receptors *ETR2* and *EIN4* were upregulated and both copies of the negative regulator *CTR1* were repressed, indicating active ethylene signalling, whereas *ACS1* was downregulated—signaling competence without the autocatalytic burst of climacteric fruit. The ABA branch responded more strongly. *NCED2* rose almost fourfold to become the most abundant hormone-related transcript, with *CCD4*, *ZEP* and both *PSY* genes upregulated and four clade-A PP2C repressors coordinately downregulated. These are the targets of the ABA-responsive module CsERF110–CsERF53 [26], while *CCD4*, which yields the peel pigment β-citraurin, is induced downstream of ethylene via a CsERF25–CsRP1 cascade [25]; *NCED* expression in the peel likewise parallels ABA accumulation during maturation [24]. Pigment formation, ABA metabolism and the color break thus form a single module, consistent with the enrichment of carotenoid biosynthesis in three of our four comparisons. This hormonal shift appears transcriptionally coordinated with flavonoid metabolism: *ACO1* and *NCED2* correlated positively with *CHI3* and *FLS*, and *CCD4* negatively with *FLS*, pairing apocarotenoid pigment formation with reduced flavonol synthase expression in agreement with the decline of flavonol and flavone glycosides as methoxylated products accumulated in the zest. A basis for such coordination exists in citrus, where AP2/ERF members activate the type IV chalcone isomerase *CitCHIL1* [27] and *CsMYBF1* regulates flavonol biosynthesis [28]. As AP2/ERF proteins are themselves hormone-responsive, we propose that ERF and MYB factors coordinate the tissue-dependent flavonoid reprogramming reported here. This remains a hypothesis from co-expression across four sample groups; hormone treatments, direct hormone quantification and promoter-binding assays are needed to test it.

### 4.3. Screening of Candidate Q-Markers for Guang Foshou and Breeding Perspectives

According to the Q-marker concept introduced in 2016, an ideal Q-marker should satisfy five principal criteria: specificity to the TCM, association with pharmacological activity, analytical measurability, dynamic variation during processing and storage, and suitability for quantitative determination [37]. Based on the spatiotemporal metabolomic data obtained in this study, a group of candidate Q-markers was identified. This included hesperidin, neohesperidin, and naringin, which were enriched in the pith, as well as the PMFs nobiletin, tangeretin, and the methylated flavonol syringetin, which were enriched in the zest. During fruit development, hesperidin and naringin progressively increased in the pith while decreasing in the zest. Their combined abundance was higher in yellow fruit than in green fruit. Syringetin was highly enriched in the mature yellow zest and is recognized for its strong antioxidant activity. The extraction of flavonoids from finger citron has previously been optimized by ethanol reflux, and the resulting flavonoid extracts have been shown to possess significant antioxidant activity in vitro [38].

Compared with Q-markers previously reported for other citrus-derived medicinal materials, these results confirmed shared characteristics and identified features characteristic of Guang Foshou. After the introduction of the Q-marker concept, Chinese researchers have conducted many studies to identify pharmacologically relevant quality markers. In Foshou, or *Citri sarcodactylis fructus*, hesperidin and diosmin have been proposed as the main Q-markers based on plant affinity, component efficacy, and measurability [39]. The quantitative literature on Foshou provides the context for these candidates. Xia et al. characterised dried Foshou powder from seven geographical regions of southern China by UHPLC coupled with mass spectrometry and identified 38 flavonoids and 29 coumarins, finding that samples with higher levels and greater diversity of phenolic constituents showed stronger antioxidant activity, and that flavonoid composition varied across regions in a manner suggesting that interactions between genomic background and environmental conditions influence metabolic activity in Foshou [17]. Diosmin and hesperidin were detected in all seven samples at 221.4 ± 10.0 to 942.1 ± 29.3 μg/g, leading the authors to propose them as common quality indicators [17], an observation consistent with the finding that diosmin, hesperidin and neohesperidin are the principal contributors to the antioxidant activity of Foshou. Tan et al. quantified 26 flavonoids in Jinhua Foshou at three developmental stages by UPLC-QqQ-MS/MS and found that total flavonoid content declined from the immature to the semimature stage and then rose to a maximum of 2360.9 ± 123.8 μg/g in mature fruit, with diosmin the most abundant flavonoid at every stage, followed by hesperidin (>500 μg/g) and rutin (>100 μg/g) [18]. Chu et al. reported diosmin and hesperidin in Guang Foshou at 1204.8 ± 1.4 and 801.6 ± 18.8 μg/g, respectively [19]. These differences in quantitative profile reflect geographical origin, developmental stage and analytical methodology, and they illustrate why markers defined on homogenised whole fruit transfer poorly between studies—the limitation that tissue-resolved markers are intended to address.

**Figure 8:**
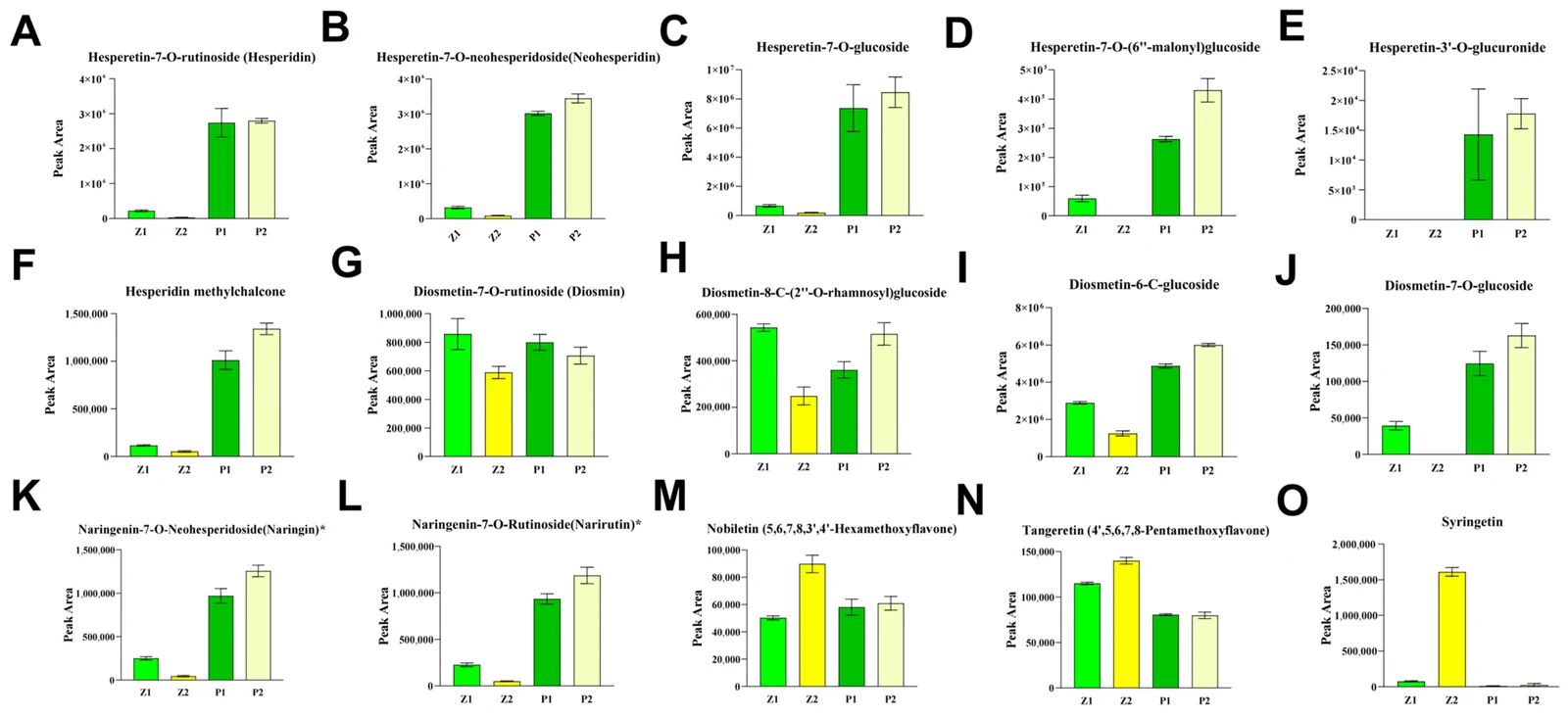
Relative abundance of candidate Q-markers in the Z1, Z2, P1 and P2 tissues of Guang Foshou. (**A**–**O**) Each panel shows one candidate Q-marker, as indicated above the panel. The y-axis gives the integrated chromatographic peak area of the corresponding MRM transition in arbitrary units (mean ± SD, *n* = 3 biological replicates). Peak areas provide a relative measure of abundance, permitting comparison of a given metabolite between tissues and developmental stages within this dataset; they are not absolute concentrations, and because the response factor of the mass spectrometer differs between compounds, the peak areas of different metabolites are not quantitatively comparable with one another. Hesperidin (**A**), neohesperidin (**B**), hesperetin-7-O-glucoside (**C**), hesperetin-7-O-(6″-malonyl)glucoside (**D**), hesperetin-3′-O-glucuronide (**E**), hesperidin methylchalcone (**F**), diosmetin-6-C-glucoside (**I**), diosmetin-7-O-glucoside (**J**), naringin (**K**) and narirutin (**L**) were more abundant in the pith than in the zest, and each increased in the pith from the green to the yellow stage, whereas diosmin (**G**) and diosmetin-8-C-(2″-O-rhamnosyl)glucoside (**H**) were of comparable abundance in the two tissues, both declining modestly from the green to the ripe stage (diosmin, −22% pooled across tissues, q = 0.0029; Table S2). Tangeretin (**N**) and syringetin (**O**) were more abundant in the zest at both stages, syringetin showing a pronounced increase in the zest at the yellow stage, whereas nobiletin (**M**) became more abundant in the zest only at the yellow stage. Z1, zest of green fruit; Z2, zest of ripe yellow fruit; P1, pith of green fruit; P2, pith of ripe yellow fruit. Naringin (**K**) and narirutin (**L**) share the transition *m*/*z* 579 → 271 and were recorded at the same retention time, so the two panels derive from a single chromatographic peak; the coefficients of variation for (**D**,**J**) across the quality-control injections were 30.5% and 44.0% (Table S1B), and these two metabolites are shown for completeness rather than proposed as markers.

In this study, hesperidin was more abundant in the pith than in the zest. Neohesperidin, hesperetin-7-O-glucoside, hesperetin-7-O-(6″-malonyl)-glucoside, hesperetin-3′-O-glucuronide, hesperidin methylchalcone, diosmetin-6-C-glucoside, diosmetin-7-O-glucoside, naringin, and narirutin were enriched in the pith (Figure 8A–L). Hesperetin-3′-O-glucuronide (Figure 8E) was the only one of these metabolites that was not detected in any zest sample at either developmental stage. In comparison, diosmin and diosmetin-8-C-(2″-O-rhamnosyl) glucoside were of comparable abundance in both tissues (Figure 8G,H). Diosmin did not differ between the zest and the pith (q = 0.32) but declined by 22% from the green to the ripe stage (q = 0.0029; Table S2); the stage effect is estimated across both tissues, which is why diosmin does not appear among the differentially accumulated flavonoids in Table S3, where each tissue is tested separately. It is therefore tissue-independent rather than invariant, and serves as a baseline marker measurable on either tissue or on whole fruit provided the determination is referenced to a defined maturity stage. Of the two polymethoxylated flavones, tangeretin was more abundant in the zest at both stages (q = 4.3 × 10^−9^), whereas nobiletin became zest-enriched only on ripening (interaction q = 0.0005; Figure 8M,N). Based on previous research, this study proposes, for the first time, that syringetin (Figure 8O) and 5-demethylnobiletin may serve as finger-citron-specific markers with both developmental-stage and tissue specificity. Syringetin is monitored through a transition, *m*/*z* 345 → 330 in negative mode, that no other compound in Table S1B shares, so its annotation does not depend on chromatographic resolution of isomers; 5-demethylnobiletin shares the transition *m*/*z* 389 → 359 with 3′-demethylnobiletin and is therefore secure as a demethylnobiletin but tentative as to the position of the free hydroxyl. These markers address an important limitation of existing quality standards, which generally focus on individual compounds or homogenized whole-fruit samples. Therefore, these results provide a direct scientific basis for establishing a multi-index quality-control system for Guang Foshou. In the case of breeding, Lin et al. reviewed advances in the synthetic biology of flavonoids. They highlighted that modification of key enzymatic genes, including *CHS*, *CHI*, *FLS*, and *F3H*, can directionally alter flavonoid composition [40]. When combined with the tissue-dependent gene-expression patterns identified in this study, these results support the application of marker-assisted breeding. Germplasm with high *CYP75B2* expression in the zest may be selected to increase PMF accumulation, whereas germplasm with high *CHS* and *CHI* expression in the pith may be selected to enhance flavanone glycoside accumulation. CRISPR/Cas9 technology may also be used to knock out enzyme-coding genes in competing metabolic pathways, promoting the accumulation of target metabolites and increasing flavonoid abundance in fruit tissues. Moreover, integrating rapid Q-marker detection methods to identify relevant marker compounds could shorten the breeding cycle by enabling breeders to conduct selection at early developmental stages rather than waiting until complete fruit maturation and ripening. These strategies provide accurate pathways for developing new Guang Foshou cultivars with enhanced medicinal value.

Two limitations qualify these markers. First, the annotations rest on MRM transitions acquired at unit mass resolution, and 132 of the 269 flavonoids share a nominal transition, in the same ionisation mode, with at least one other library entry. Diosmin, nobiletin and syringetin each occupy a transition that no other entry shares, so neither tier of the framework depends on resolving isomers. Naringin and narirutin, however, share *m*/*z* 579 → 271 at the same retention time and give near-proportional profiles across the twelve samples (r = 0.99), so they represent one chromatographic peak rather than two markers; and tangeretin, 5-demethylnobiletin, hesperidin and neohesperidin each share a transition with one to three library isomers, making these annotations secure as isomer classes but tentative as individual isomers. Methods derived from them should be validated against authentic standards, co-eluting isomers being quantified as a summed signal. Second, the green-fruit samples of both tissues were injected in the first block and the ripe-fruit samples in the second (Table S1A), so developmental stage is confounded with analytical batch; the zest-versus-pith comparisons, on which the principal conclusions rest, were made within blocks and are unaffected. The quality-control injections bracketing the sequence bound the batch contribution: the median ratio of the last to the first was 0.97, and only 35.7% of metabolites changed monotonically across the three injections against 33.3% expected by chance, indicating variation without systematic direction. The median stage difference, 2.1-fold in the zest and 1.6-fold in the pith, is well above this bound, but the smallest significant differences, 14% to 17%, are of the same order as the analytical variation and should not be interpreted individually; confirming the stage-dependent markers would require an independent sample set analysed in randomised injection order.

## 5. Conclusions

The combined metabolomic and transcriptomic analyses characterized the spatiotemporal patterns of flavonoid accumulation in the pith and zest of Guang Foshou (*Citrus medica* var. *sarcodactylis*) at different developmental stages. The results showed that the transition from green to yellow fruit was associated with tissue-dependent metabolic changes. The yellow zest preferentially accumulated polymethoxylated flavones (nobiletin, tangeretin) and the methylated flavonol syringetin, which possess anti-inflammatory, anticancer, and neuroprotective activities. However, the yellow pith was enriched in flavanone glycosides (hesperidin, neohesperidin, and naringin), which are associated with cardioprotective and antioxidant effects. This distribution of chemical components provides a scientific basis for the traditional quality criterion of “yellow zest with white pith.” Moreover, stage-dependent markers were identified, including C-glycosylflavones in green fruit, polymethoxylated flavones in yellow zest, and flavanone glycosides in yellow pith. These compounds may serve as objective indicators for harvest timing and quality control. Diosmin, indistinguishable between the two tissues, can serve as the baseline tier for whole-fruit or single-tissue material, referenced to a defined maturity stage since its abundance fell by about one fifth on ripening. The main biosynthetic genes *CHS*, *CHI*, *FLS*, *CYP75B2*, and *FGT* showed tissue- and stage-dependent expression patterns consistent with their respective metabolite profiles, indicating their potential as candidate targets for marker-assisted breeding. And the coordinated upregulation of ethylene and abscisic acid biosynthetic genes in the zest, together with their co-expression with flavonoid structural genes, indicates that the tissue-dependent flavonoid pattern is embedded in the hormonal programme of the green-to-yellow transition. Thus, this study enhances understanding of flavonoid diversification in finger citron and provides a practical basis for evaluating medicinal quality and molecular breeding of Guang Foshou.

## Data Availability

The raw RNA-sequencing data generated in this study have been deposited in the Genome Sequence Archive (GSA) of the National Genomics Data Center (NGDC), China National Center for Bioinformation, under accession number CRA052375, and are accessible at https://ngdc.cncb.ac.cn/gsa/browse/CRA052375 (accessed on 21 October 2026). The metabolite abundance table underlying all metabolomic analyses has been deposited in the Open Archive for Miscellaneous Data (OMIX) of the same centre under accession number OMIX022528, accessible at https://ngdc.cncb.ac.cn/omix/release/OMIX022528 (accessed on 21 October 2026). All other data supporting the findings of this study are provided in the manuscript and its Supplementary Materials.

## Supplementary Materials

The following supporting information can be downloaded at https://www.mdpi.com/article/10.3390/foods15213946/s1, Figure S1: Classification of annotated metabolites and PCA transcriptome analysis in the pith and zest of green and yellow Guang Foshou fruits. (A) Percentage distribution of 11 detected metabolite classes in each fruit sample. (B) Principal component analysis (PCA) plot showing transcriptomic differences among P1, Z1, P2, and Z2. Figure S2: The four-group PLS-DA model gave a leave-one-out Q^2^ of 0.516, exceeding all but one of 999 permutations (*p* = 0.002). The pairwise OPLS-DA models gave leave-one-out Q^2^ values of 0.955 to 0.998, each exceeding all nine alternative partitions (−0.89 to −0.41), though with only ten partitions available the exact *p*-value cannot fall below 0.10. R^2^Y is reported for completeness; with 269 variables and six samples per comparison it is not an informative measure of model quality. Figure S3: Permutation testing of the multivariate models. (A–D) Leave-one-out Q^2^ of all ten partitions of the six samples in each pairwise comparison: (A) P1 vs. Z1, (B) P2 vs. Z2, (C) Z1 vs. Z2, (D) P1 vs. P2. The observed partition is at “obs” and the nine alternatives at 1–9; all ten were enumerated rather than resampled, so the smallest attainable exact *p*-value is 0.10. (E) Null distribution of the leave-one-out Q^2^ of the four-group PLS-DA model over 999 random permutations, the observed value marked by the vertical line. No regression was fitted and no intercept is reported. Figure S4: GO and KEGG enrichment analysis of differentially expressed genes among the four tissue groups. (A–D) GO enrichment of the differentially expressed genes in P1 vs. P2, Z1 vs. Z2, P1 vs. Z1 and P2 vs. Z2Z2, respectively, showing the biological process, cellular component and molecular function categories. (E–H) KEGG pathway enrichment for the same four comparisons, in the same order. Table S1A: Injection sequence of the widely-targeted metabolomic analysis. Table S1B: Flavonoid metabolites identified in the zest and pith of Guang Foshou at the green and ripe yellow stages. Table S2: Differentially accumulated flavonoids (DAFs) in each pairwise comparison of the zest and pith of Guang Foshou at the green and ripe yellow stages. Table S3: Two-way analysis of variance of individual flavonoids in the zest and pith of Guang Foshou at the green and ripe yellow stages. Table S4: Sequencing, filtering and alignment statistics for the twelve RNA-sequencing libraries.

## Abbreviations

The following abbreviations are used in this manuscript:

TFC	Total flavonoid content
DAFs	Differentially accumulated flavonoid metabolites
PCA	Principal component analysis
PLS-DA	Partial Least Squares-Discriminant Analysis
VIP	Variable Importance in the Projection
FPKM	Fragments per kilobase of transcript per million mapped reads
DEGs	Differentially expressed genes
KEGG	Kyoto Encyclopedia of Genes and Genomes
PMFs	Polymethoxylated flavones
UHPLC	Ultra-high-performance liquid chromatography
TCM	Traditional Chinese medicine
Q-marker	Quality-marker
FDR	False discovery rate
MRM	Multiple reaction monitoring
QTRAP	Quadrupole-linear ion trap
LIT	Linear ion trap
QQQ	Triple quadrupole
